# Dopaminergic and Opioid Pathways Associated with Impulse Control Disorders in Parkinson’s Disease

**DOI:** 10.3389/fneur.2018.00109

**Published:** 2018-02-28

**Authors:** Aleksander H. Erga, Ingvild Dalen, Anastasia Ushakova, Janete Chung, Charalampos Tzoulis, Ole Bjørn Tysnes, Guido Alves, Kenn Freddy Pedersen, Jodi Maple-Grødem

**Affiliations:** ^1^The Norwegian Centre for Movement Disorders, Stavanger University Hospital, Stavanger, Norway; ^2^Department of Research, Section of Biostatistics, Stavanger University Hospital, Stavanger, Norway; ^3^Department of Neurology, Haukeland University Hospital, Bergen, Norway; ^4^Department of Clinical Medicine, University of Bergen, Bergen, Norway; ^5^Department of Neurology, Stavanger University Hospital, Stavanger, Norway; ^6^Department of Mathematics and Natural Sciences, University of Stavanger, Stavanger, Norway; ^7^The Centre for Organelle Research, University of Stavanger, Stavanger, Norway

**Keywords:** Parkinson’s disease, impulse control disorders, addiction, elastic net, OPRK1, DRD1

## Abstract

**Introduction:**

Impulse control disorders (ICDs) are frequent non-motor symptoms in Parkinson’s disease (PD), with potential negative effects on the quality of life and social functioning. ICDs are closely associated with dopaminergic therapy, and genetic polymorphisms in several neurotransmitter pathways may increase the risk of addictive behaviors in PD. However, clinical differentiation between patients at risk and patients without risk of ICDs is still troublesome. The aim of this study was to investigate if genetic polymorphisms across several neurotransmitter pathways were associated with ICD status in patients with PD.

**Methods:**

Whole-exome sequencing data were available for 119 eligible PD patients from the Norwegian ParkWest study. All participants underwent comprehensive neurological, neuropsychiatric, and neuropsychological assessments. ICDs were assessed using the self-report short form version of the Questionnaire for Impulsive-Compulsive Disorders in PD. Single-nucleotide polymorphisms (SNPs) from 17 genes were subjected to regression with elastic net penalization to identify candidate variants associated with ICDs. The area under the curve of receiver-operating characteristic curves was used to evaluate the level of ICD prediction.

**Results:**

Among the 119 patients with PD included in the analysis, 29% met the criteria for ICD and 63% were using dopamine agonists (DAs). Eleven SNPs were associated with ICDs, and the four SNPs with the most robust performance significantly increased ICD predictability (AUC = 0.81, 95% CI 0.73–0.90) compared to clinical data alone (DA use and age; AUC = 0.65, 95% CI 0.59–0.78). The strongest predictive factors were rs5326 in *DRD1*, which was associated with increased odds of ICDs, and rs702764 in *OPRK1*, which was associated with decreased odds of ICDs.

**Conclusion:**

Using an advanced statistical approach, we identified SNPs in nine genes, including a novel polymorphism in *DRD1*, with potential application for the identification of PD patients at risk for ICDs.

## Introduction

Patients with Parkinson’s disease (PD) have a threefold increased odd for developing impulse control disorders (ICDs) and related compulsive behaviors when compared to controls (1, 2). These behaviors are characterized by lacking control of rewarding behaviors, such as gambling, sexual activity, eating, and buying. In addition, patients may also develop a preoccupation with hobbies, punding behaviors, and an addiction-like pattern of dopaminergic medication use. Although common in PD, ICDs are not merely a result of PD pathology (3), but are closely associated with the use of dopaminergic replacement therapy (DRT), such as dopamine agonists (DAs) (1, 2, 4). Still, not all patients develop ICDs when exposed to dopaminergic medications, arguing that some individuals are more susceptible to DRT than others. Previously identified demographic-risk factors, such as familial history of addiction, increased impulsivity, and novelty-seeking traits (1, 5), argue that the individual vulnerability may be of genetic origin.

To date, the evaluation of ICD susceptibility in PD has primarily focused on independent associations of single genetic variants. Several studies have reported an association between ICD development in PD patients and genetic polymorphisms in dopamine receptor (*DRD1–3*) and glutamate receptor (*GRIN2B*) genes (6–9), while individual studies also point toward a potential association with genetic polymorphisms in serotonin receptor (*HTR2A*), dopamine transporter (*DAT1*), and tryptophan hydroxylase 2 (*TPH2*) genes (10, 11). Recently, the spectrum of monoaminergic ICD candidate genes was expanded through the identification of a polymorphism in *OPRK1*, which encodes an opioid receptor, as the strongest genetic predictive factor in a clinical–genetic model designed to predict the occurrence of ICDs in early PD in the Parkinson’s Progression Markers Initiative (PPMI) cohort (12). The authors further reported that the inclusion of a panel of candidate-genetic variants improved the prediction of incident ICDs (identifying up to 76% of incident ICD cases in early-stage PD patients) compared to prediction based on clinical variables alone (12), arguing for the potential clinical utility of genetic testing. The authors estimated that common genetic variants accounted for 57% of the variance of ICD incidence among PD patients in the PPMI study. This heritability estimate is comparable to estimates from the general population, but current knowledge about individual risk genes is limited. We suggest that several neurotransmitter systems may contribute to ICD pathogenesis, and multiple genes within one system may play a crucial role in the pathogenesis of these behaviors.

To date, the identification of patients at risk of ICDs remains a primary aim in clinical research. Although several genetic polymorphisms have been suggested to aid clinical identification of ICD risk, most published studies utilize a candidate-gene approach based on previously published findings. In this study, we aimed to determine the association of genetic polymorphisms across several neurotransmitter pathways using an advanced statistical approach. A secondary aim was to investigate the clinical utility of a genetic panel in the prediction of ICD status in patients with PD.

## Materials and Methods

### Study Design

This cross-sectional study is based on participants from the Norwegian ParkWest study, a population-based longitudinal study of incident PD. The ParkWest cohort is composed of patients with newly diagnosed PD and normal control subjects recruited from four counties in Norway between 2004 and 2006, who were prospectively followed up by movement disorder neurologists. A detailed presentation of the diagnostic procedures and case ascertainment has previously been published (13). Screening for ICDs was first introduced at 5-year follow-up, and this study included 155 patients with PD who still remained in the study after 5 years of follow-up. Of these, 28 patients were excluded due to dementia and two due to missing data on Questionnaire for Impulsive-Compulsive Disorders in Parkinson’s Disease (QUIP), leaving 125 patients eligible for this study. Patients with missing information on relevant genetic variants (*n* = 6) were removed from this study.

### Clinical Measures

A standardized examination program was administered by trained members of the ParkWest study group. Information regarding demographic variables, lifestyle factors, clinical history, and medication was obtained using semi-structured interviews. Severity of motor symptoms was assessed using the Unified Parkinson’s Disease Rating Scale (UPDRS) part III (14). Self-evaluated functioning on activities of daily life and complications of dopaminergic therapy were assessed using UPDRS parts II and IV. Hoehn and Yahr (H&Y) was used to assess disease stage (15). Levodopa equivalent doses (LEDs) were calculated according to published recommendations (16). Mini-Mental State Examination (MMSE) was used to assess global cognitive functioning (17). The Montgomery and Aasberg Depression Rating Scale (MADRS) was used to assess depressive symptoms (18). Lastly, ICDs were assessed using the self-report short form version of the QUIP (19). Participants with a positive response to one or more screening questions of the QUIP were classified to have ICD (20).

### Candidate Gene and Variant Selection

Of the 125 patients eligible for this study, 119 had previously been characterized by whole-exome sequencing (WES) (unpublished material). We selected 16 genes (*ADRA2C, DRD1–5, SLC6A3/DAT1, DDC, COMT, SLC6A4/5HTTLPR, TPH2*, HTR2A, OPRM1, OPRK1, *GRIN2B*, and *BDNF)* based on established roles in candidate neurotransmitter pathways, or a published involvement in ICD and related behaviors in either patients with PD or in non-PD populations. This was achieved by performing a literature search, and the genes identified were involved in four neurotransmitter pathways (dopaminergic, serotonergic, glutamatergic and opioid) (6–12). All variants (*n* = 185) present in the candidate-gene regions were extracted using ingenuity variant analysis (Qiagen, CA, USA) and filtered to retain only those with minor allele frequency (MAF) >0.5 in the ParkWest and the 1,000 genomes project (*n* = 71). A further 12 single-nucleotide polymorphisms (SNPs) were removed based on a high linkage disequilibrium (LD) measured using the Broad Institute SNP Annotation and Proxy Search (SNAP) (21). In addition, two SNPs that have frequently been studied in ICDs in PD, but which were not in the original data extraction, were also included: rs1800497 in *ANKK1* was extracted from the WES data and rs6280 in *DRD3* was genotyped using a custom-made TaqMan SNP-genotyping assay (Thermo Fisher Scientific), as described (22). For further analysis, the genotypes were converted to carrier status, and five variants removed due to a carrier frequency >95% in the study population.

### Statistical Analyses

Statistical procedures were performed using IBM SPSS Statistics version 24.0.0.1, R 3.4.0 and STATA IC 14.2. Group differences were analyzed using *t*-tests, Mann–Whitney tests, χ^2^–tests, and Fisher exact tests as appropriate.

Performing an extensive investigation of genetic variants associated with ICDs is inherently difficult due to the large number of possible variants identified in a single neurotransmitter pathway. The number of variants (*p)* will often exceed the number of participants (*n*) in the study. In these cases (*p* >> *n*), the traditional strategies for multivariable regression modeling will fail. An option here is to assume a sparse solution, i.e., that only a small subset of variants are involved in a single neurotransmitter pathway. Recent advances in statistical modeling, such as elastic net (EN) regularized generalized linear regression, reduce the number of predictors by penalizing those that do not have enough prediction power. This allows one to reduce the risk of overfitted models and increase the generalizability to other cohorts (23, 24). In this study, regularized logistic regression with EN penalization was used to identify SNPs associated with ICDs. Regularized regression with EN is well suited for model selection of high-dimensional data, as is often the case in analyses of genetic polymorphisms in clinical cohorts (23, 25). In addition, EN handles variants with high LD and multiple SNPs from one neurotransmitter pathways well (26).

Elastic net analyses were performed in *R*, using the *glmnet*-package (27). The level of regularization parameter λ was chosen as the minimal λ that yielded prediction error estimated by cross-validation within one standard error from its minimal value. In the *glmnet*, the parameter α decides the balance between l_1_ and l_2_ regularizations, of which the former is the regularization used in Lasso regression (α = 1) and the latter is used in Ridge regression (α = 0). In our analyses, the EN was repeated for all α from 0 to 1, with 0.01 increments. Non-zero estimated coefficients consistent throughout the entire range of α support the evidence of associations between relevant SNPs and ICD status.

**Figure 1:**
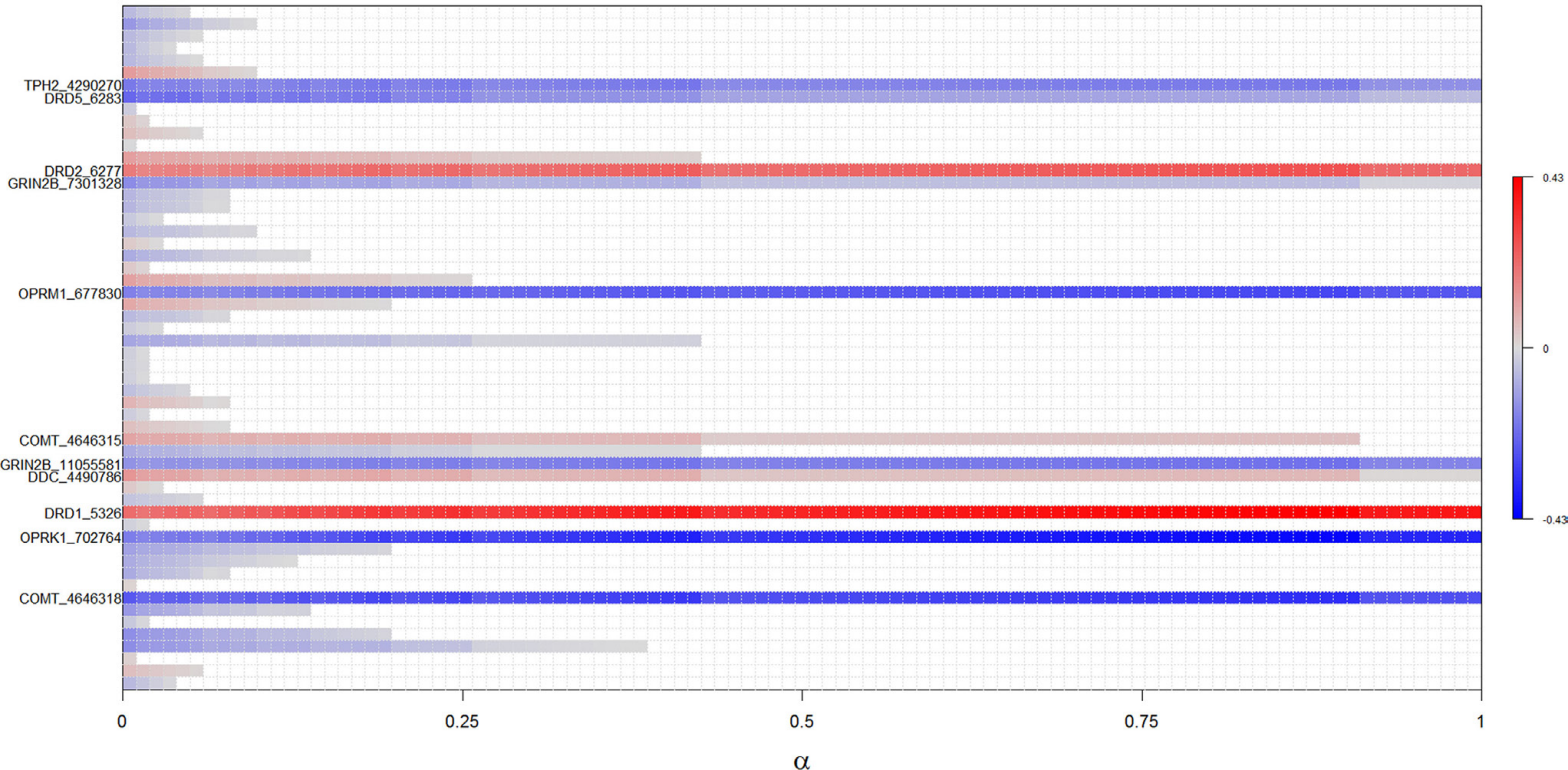
Results of regularized regression with elastic net penalization for α-values between 0 and 1. Polymorphisms positively associated with ICDs (i.e., increases risk) are highlighted with red, while polymorphisms negatively associated with ICDs (i.e., decreases risk) are highlighted in blue, with the intensity of color reflecting the strength of association. Polymorphisms not associated with ICDs are white. Identified polymorphisms demonstrate significant association across all levels of α.

The discriminative ability of the biomarkers with regard to ICD diagnosis was assessed from receiver-operating characteristic (ROC) curve analysis. The test variable was the predicted probability from logistic regression with ICD diagnosis (yes/no) as outcome. In order to not overfit the model, the four SNPs with a most robust performance in EN analysis were selected as candidate SNPs. Robustness of candidate SNPs was defined by the consistency of the estimated *B*-values in EN analyses (which are visually represented by color in Figure 1). The ROC curve was plotted with preselected clinical variables alone (age and either DA use), for the genetic variables alone (genetic model), and with the clinical and candidate SNP data combined (clinical–genetic model). Area-under-the-curve (AUC) values were compared using DeLong test.

## Results

### Demographic and Clinical Characteristics

Demographic and clinical characteristics are presented in Table 1. Of 119 patients in the study, 29.4% (35/119) reported at least one ICD. Patients with ICD did not differ from patients without ICD in terms of sex, education, duration of PD, MMSE scores, or scores on UPDRS II, III, or IV, but patients with ICDs tended to be younger (*p* = 0.050) and scored significantly higher on MADRS (*p* = 0.010). Patients with ICDs also used DA more frequently (*p* = 0.001) and had a higher total LED (*p* = 0.017). DA dosage was not different when comparing DA users with ICDs with those without ICDs (*p* = 0.958).

**Table 1:** Demographic and clinical characteristics.

Characteristics	Total (*n* = 119)	ICD (*n* = 35)	No ICD (*n* = 84)	*p*-Value^a^
Age	70.5 (9.3)	67.9 (7.7)	71.6 (9.7)	**0.050**
Male, *n* (%)	74 (62.2)	25 (71.4)	49 (58.3)	0.180
Education	11.6 (3.2)	11.49 (3.0)	11.7 (3.3)	0.803
Duration of PD	7.4 (1.8)	7.3 (1.4)	7.4 (1.9)	0.658
Mini-Mental State Examination	27.8 (2.6)	28.5 (1.7)	27.5 (2.8)	0.063
Montgomery and Aasberg Depression Rating Scale	3.9 (4.4)	5.5 (5.1)	3.2 (4.0)	**0.010**
UPDRS II	10.7 (5.4)	12.0 (6.0)	10.1 (5.0)	0.126
UPDRS III	22.7 (10.8)	23.8 (10.7)	22.3 (10.9)	0.422
UPDRS IV	1.8 (1.7)	2.0 (1.8)	1.7 (1.7)	0.369
Hoehn and Yahr stage	2.2 (0.6)	2.2 (0.6)	2.2 (0.6)	0.920
DA users, *n* (%)	75 (63.0)	30 (85.7)	45 (53.6)	**0.001**
Total LED	619.0 (350.2)	740.7 (354.9)	568.2 (333.7)	**0.017**

*PD, Parkinson’s disease; UPDRS, Unified PD Rating Scale; DA, Dopamine agonist; LED, Levodopa equivalent dosage; ICD, Impulse control disorder*.

*^a^Group differences between patients with and without ICDs*.

*Significant p-values are highlighted in bold*.

### Variant Selection

The complete results from EN analyses are presented in Figure 1. Fifty-six SNPs were identified across the genes selected for analysis (Table S1 in Supplementary Material), and 11 SNPs from four neurotransmitter pathways were robustly associated with ICDs across all levels of α in the EN analysis (Figure 1; Table 2). Specifically, carriers of the minor alleles of the *DRD1* rs5326, *DRD2* rs6277, COMT rs4646315, and DDC rs4490786 SNPs were associated with an increased risk of ICDs. Carriers of the minor allele of the *OPRM1* rs677830, *OPRK1* rs702764, *GRIN2B* rs1105581 and rs7301328, *COMT* rs4646318, TPH2 rs4290270, DRD5 rs6283 SNPs were associated with a decreased risk of ICDs. Of these, the *DRD1* rs5326, *OPRK1* rs702764, *OPRM1* rs677830, and *COMT* rs4646318 were most robustly associated with ICD status and thus considered candidate variants.

**Table 2:** Characteristics of identified SNPs in elastic net analysis.

					MAF^c^		
Gene	SNP	Location^a^	Transcript^b^	Protein	ParkWest	1,000 genomes	Association with impulse control disorders in ParkWest^d^
DRD1	rs5326	5:175443193	c.-94G > A		0.14	0.17	+
DRD2	rs6277	11:113412737	c.957C > T	p.Pro319Pro	0.50	0.24	+
OPRM1	rs677830	6:154107531	c.1231C > T	p.Gln411Ter	0.29	0.15	−
OPRK1	rs702764	8:53229597	c.843A > G	p.Ala281Ala	0.11	0.24	−
GRIN2B	rs11055581	12:13675725	c.1125 + 20A > G		0.18	0.10	−
COMT	rs4646318	22:19967324	c.466 − 1212G > A		0.07	0.07	−
TPH2	rs4290270	12:72022455	c.1125A > T	p.Ala375Ala	0.64	0.49	−
DRD5	rs6283	4:9783007	c.978C > T	p.Pro326Pro	0.60	0.39	−
GRIN2B	rs7301328	12:13865843	c.366C > G	p.Pro122Pro	0.46	0.44	−
DDC	rs4490786	7:50476616	c.1041 + 8G > A		0.18	0.20	+
COMT	rs4646315	22:19964374	c.615 + 75G > C		0.19	0.17	+

*SNPs, single-nucleotide polymorphisms; MAF, minor allele frequency*.

*^a^Genome location in GRCh38 assembly*.

*^b^Transcript position of most severe consequence according to the Human Genome Variation Society guidelines (28)*.

*^c^MAF in the patients of the ParkWest cohort or 1,000 genomes project*.

*^d^“+” indicated a positive association with ICDs in the ParkWest cohort and “−” indicates a negative association with ICDs in the Park cohort*.

### Prediction of ICDs

**Figure 2:**
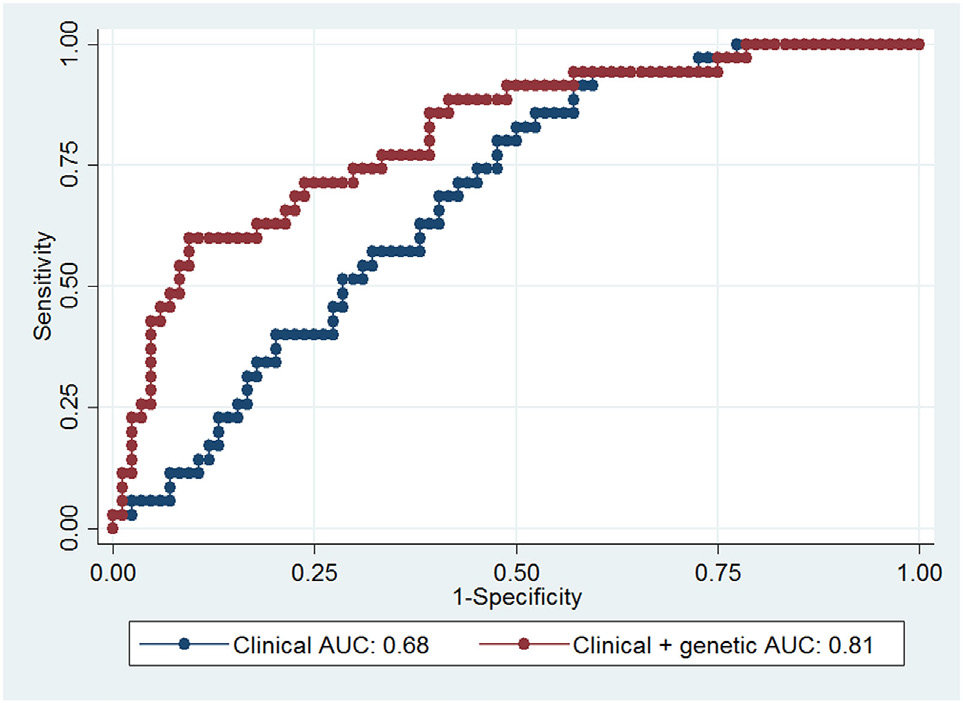
Receiver-operating characteristic (ROC) curves for prediction of impulse control disorders (ICDs). The blue curve was plotted with clinical variables (age and dopamine agonist use), while the red curve was plotted with clinical and the four candidate single-nucleotide polymorphisms. Area under the curve (AUC) for each model is indicated in the figure.

The prediction of ICDs was estimated by using ROC curves with AUC (Figure 2). In the clinical model, ROC curves plotted with the clinical variables age and DA use yielded an estimated AUC of 0.68 (95% CI 0.59–0.78). In this analysis, DA use [odds ratio (OR) 4.5; 95% CI 1.5–13.5; *p* = 0.006] was associated with the presence of ICDs. The genetic model, consisting of the SNPs *DRD1* rs5326, *OPRK1* rs702764, *OPRM1* rs677830, and *COMT* rs4646318, yielded an estimated AUC of 0.70 (95% CI: 0.61–0.79). Of these, one variant, the *DRD1* SNP rs5326, was significantly associated with ICDs (OR 2.9; 95% CI 1.1–7.6; *p* = 0.026).

In the clinical–genetic model, we included four candidate SNPs identified in the EN analyses, resulting in an estimated AUC of 0.81 (95% CI 0.73–0.90). This 13% point increase in AUC between the clinical and the clinical–genetic model was statistically significant (*p* = 0.003). Similarly, the 11% point increase in AUC between the genetic and the clinical–genetic model was also significant (*p* = 0.008). In the clinical–genetic model, DA use (OR 7.4; 95% CI 2.1–26.2; *p* = 0.002) was again associated with increased odds of ICDs, and the significant genetic predictors *DRD1* SNP rs5326 (OR 6.1; 95% CI 1.9–19.6; *p* = 0.003) and *OPRK1* SNP rs702764 (OR 0.2; 95% CI 0.1–0.8; *p* = 0.040) were associated with an increased and a decreased risk of ICDs, respectively. Full details of the clinical and the clinical–genetic models are presented in Table 3.

**Table 3:** Association between ICD status and a clinical, genetic, and clinical + genetic model.

	Clinical model		Genetic model		Clinical + genetic model	
Factor	OR (95% CI)	*p*-Value^a^	OR (95% CI)	*p*-Value^a^	OR (95% CI)	*p*-Value^a^
(Intercept)	0.6	0.756	0.1	0.099	1.1	0.948
Age	1.0 (0.9–1.0)	0.434	–	–	1.0 (0.9–1.0)	0.234
DA use	4.5 (1.5–13.5)	**0.006**	–	–	7.4 (2.1–26.2)	**0.002**
DRD1 rs5326	–	–	2.9 (1.1–7.6)	**0.026**	6.1 (1.9–19.6)	**0.003**
OPRK1 rs702764	–	–	0.3 (0.1–1.1)	0.072	0.2 (0.1–0.9)	**0.040**
OPRM1 rs677830	–	–	0.5 (0.2–1.2)	0.105	0.5 (0.2–1.3)	0.153
COMT rs4646318	–	–	0.3 (0.1–1.5)	0.140	0.2 (0.1–1.5)	0.117

*OR, odds ratio; 95% CI, 95% confidence interval; DA, dopamine agonist; ICD, impulse control disorder*.

*^a^Single factor association from stepwise logistic regression with ICD status as dependent variable*.

*Significant p-values are highlighted in bold*.

## Discussion

In this study, we identified an association between ICDs and SNPs in the dopaminergic, glutamatergic, serotonergic, and opioid neurotransmitter system using an advanced statistical procedure. Using four polymorphisms from this panel significantly increased the level of prediction of ICD status beyond known clinical risk factors. These results confirm and expand existing knowledge about the genetic architecture of ICDs in PD. To date, this is the most extensive investigation of polymorphisms in relation to ICDs in PD.

### Guiding Clinical Practice Using Genetic Markers

Despite new insights into the pathophysiology of ICDs in PD, a consistent model for clinical differentiation between patients with high and low risk of ICDs has still not been developed. Although younger age has been associated with ICDs in several cohorts, DA is more often prescribed to younger patients than that to older. As evident in the clinical model of ICD risk, age is not significantly associated with ICDs when controlling for DA use (Table 3). Even though DA use is the predominant risk factor for ICDs in patients with PD, DA is still a preferred drug in the early stages of PD due to the diminishing effects of levodopa over time. Therefore, the identification of risk factors that predict ICDs *before exposure* to DA is important to guide clinical practice. Genetic panels have been advocated to be a clinically useful predictor of disease and may be especially important when investigating common polymorphisms, which may have a small effect size and be contingent upon gene-by-environment interactions. Recently, a predictive genetic panel for ICDs in PD has been proposed. Kraemmer and colleagues utilized a panel of 13 candidate polymorphisms, which in concert with clinical variables resulted in an AUC of 76% (95% CI 70–83%) for prediction of ICDs. Our findings support the use of a genetic and clinical model in the prediction of ICDs in PD and also advocate for an approach in which genetic variants are selected based on not only the previously published literature but also using a statistical approach that can handle a gamut of variants. Using such an approach, we have replicated the finding that OPRK1 rs702764 is associated with ICDs when controlling for DA use and identified a novel association between an SNP in *DRD1* and ICDs. In addition, we also identified a sparse clinical–genetic model with a high degree of prediction [AUC of 81% (95% CI 73–90%)] of ICD status, using only four candidate SNPs.

### Dopaminergic Pathways

When controlling for DA use and age, we identified two genes with polymorphisms that were independently associated with ICDs (Table 3). rs5326 is positioned in the 5′ untranslated region (UTR) of the *DRD1* gene, which encodes the dopamine receptor D1, and was associated with an increased risk of ICDs. The D1 receptor is the most abundant dopamine receptor in the central nervous system, particularly expressed in the prefrontal areas, and is considered a modulator of dopaminergic activity (29). Stimulation of D1 receptors by agonists or illicit drugs (like cocaine and amphetamine) has been suggested to trigger punding and hobbyism behaviors in both patients with PD and patients with addiction (30). Previously, polymorphisms in the noncoding regions of *DRD1* (rs4867798 in the 3'-UTR and rs4532 in the 5'-UTR) have been associated with ICDs in a Malaysian PD cohort (8). Furthermore, polymorphisms in *DRD1* have been linked to ICDs, neuropsychiatric disease, problem gambling, addiction, and cognitive functioning in non-PD populations (31, 32). Risk variants of rs5326 have been associated with a decreased *DRD1* expression, a reduced cognitive functioning in both healthy males and bipolar patients, and an increased risk of neuropsychiatric disorders, such as schizophrenia and heroin addiction (33–36).

Few studies have investigated the *DRD1* gene with regard to ICDs in PD, while considerable effort has been made in identifying polymorphisms in *DRD2* and *DRD3*, mostly due to the established importance of these genes in ICDs in the general population and the high affinity of DAs to these receptors (37, 38). In our data, the rs6277 SNP in *DRD2* was robustly associated with ICDs in the EN analysis, but was not a strong individual predictor of ICD in regression analysis. rs6277 has previously been associated with individual differences in cognitive functioning, reward processing, and impulsivity (39–45). Although the association between ICDs and the rs6277 is novel, it should be noted that this SNP has not been included in previous studies of ICDs in PD. Several other genetic variants in *DRD2*, including rs6277 neighboring SNP rs1800497 (Taq1A), have been studied in PD and found to be associated with ICDs, although not in all studies (6–8, 12).

The D1 and D2 receptors have been suggested to have opposing roles in reward processing, modulating reward and avoidance-based learning, respectively (46). However, the precise interplay between polymorphisms in *DRD1* and *DRD2* and the presentation of ICDs is largely unknown. One theory suggests that polymorphisms in the promoter region of *DRD1* can affect mRNA stability and result in a lower expression of the D1 receptor itself (8, 32). Given the modulating role of the *DRD1* gene in dopaminergic signaling and reward processing, patients with polymorphisms may be prone to a hyperdopaminergic state when exposed to DRT. Similarly, some authors have speculated that polymorphisms in *DRD2*, like the Taq1A polymorphism, may result in modifications in the protein structure of the receptor and ultimately lead to a reduced expression of the D2 receptor (8). This theory is supported by neuroimaging studies that have identified low D2/D3 receptor availability in ventral striatum in patients with ICDs [see (47) for a review]. However, it is still unknown if polymorphisms in these SNPs can result in a reduced expression of D1 and D2 receptors and, if so, if these polymorphisms result in functional dysfunctions, like aberrant reward processing. In order to test these theories, studies at the cellular and molecular levels are needed.

### Opioid Pathways

The second polymorphism having an independent association with ICDs was rs702764, located in the kappa-opioid receptor (*OPRK1*) gene. This polymorphism was negatively associated with ICDs in the clinical–genetic model. *OPRK1* encodes the kappa-opioid receptor 1 (KOR1), which is one of four-related opioid receptors in the brain. KOR1 is involved in processes such as feeding behavior, pain management, and addiction. In rodent models, the *OPRK1* gene has been shown to modulate dopaminergic tone, suggesting that *OPRK1* is involved in reward processing (48, 49). Previously, the TC genotype of the *OPRK1* SNP rs702764 has been associated with incident ICDs (12). The neurophysiology between KOR1 and dopamine signaling is not fully understood, but some authors have suggested that the opioid receptors mu1 (MOR1) and KOR1 have opposing roles in the modulation of basal dopaminergic tone in the nucleus accumbens (50–52). Thus, the involvement of the *OPRK1* in modifying the risk of ICDs may be of special interest due to the potential for pharmacological interventions with opioid antagonists. The opioid antagonist naltrexone, which has high affinity to the MOR1 and KOR1, has been deemed efficacious in reducing the severity of other ICDs, such as hoarding and compulsive disorders in the general population. To date, only one trial with PD patients has been published (53). Although naltrexone was not associated with change on the Clinical Global Impression scale, naltrexone was associated with significant changes in QUIP score, arguing that further studies are warranted.

The possible association between polymorphisms in dopamine and opioid receptors and ICDs is interesting, as they are also considered candidate genes for what has been termed “reward deficiency syndrome,” a hypothesized neuropsychological state characterized by decreased feelings of satisfaction caused by gene-by-environment interactions (37, 54, 55). This theory, composed of evidence from ICD patients without PD, suggests that polygenic variability, given the right environmental factors, could result in a hypodopaminergic state that causes insensitivity to reward and results in an atypical reward-seeking behavior, as often seen in patients with behavioral or chemical addictions. However, the current models of ICDs in PD suggest that ICDs in PD are a result of a hyperdopaminergic state, caused by exogenous dopamine and possibly exacerbated by frontal cognitive dysfunctions (56, 57). Based on these observations, one might argue that although ICDs in patients with PD and patients without PD are similar in terms of phenotype and share genetic risk profiles, the gene-by-environment profiles and pathophysiology might differ in the two populations.

### Strengths and Limitations

There are several limitations that should be considered. First, we have not validated our findings in an external cohort, making generalization or clinical utility of these findings impossible before replication. Despite this, our approach positively identifies variants previously associated with ICDs in the PPMI study (12) and provides new insights into the genetic architecture of ICDs in PD. A second limitation is the use of QUIP as a definition of ICDs. This measure has high sensitivity, but lacks specificity and may inflate the frequency estimates of ICDs. Third, causative relations between the identified genetic polymorphisms and ICDs are difficult to infer based on the current research design. Due to the involvement of DA in ICD development, one might argue that the identified SNPs could increase the risk of DA use, rather than ICDs. We have attempted to meet this challenge by adopting a clinical–genetic model that controls for DA use. Strengths of this study include the use of patients with and without ICDs that are matched in terms of motor impairment and H&Y stage. As argued by Cormier and colleagues, investigations into the genetic architecture of ICDs in PD should include matched groups in terms of motor impairment, H&Y stage, and DA LED (58). Although patients differed in terms of total LED, patients with ICDs were not significantly different than patients without ICDs in terms of DA LED. Lastly, we argue that using an advanced statistical approach that yields robust findings when analyzing a large amount of variants is a major strength of this study.

## Conclusion

Our findings demonstrate that a genetic panel (DRD1, OPRK1, OPRM1, and COMT) can provide valuable information with regard to the clinical differentiation between PD patients at risk of ICDs and PD patients without risk. Using an advanced statistical approach, we also identified one novel polymorphism associated with ICDs in PD. Although promising, our results need replication in other, larger cohorts.

## Ethics Statement

All subjects gave written informed consent in accordance with the Declaration of Helsinki. The protocol was approved by the Regional Committee for Medical and Health Research Ethics, Western Norway.

## Author Contributions

AE was involved in the conception, design, statistical analysis, interpretation of data, and writing of the first draft. JG and KP were involved in the conception, design, interpretation of data, and supervision of the study. ID and AU were involved in statistical analysis and interpretation of data. JG, CT, and JC were involved in the analysis of genetic data. GA and OT were involved in the conception and study supervision. All authors made critical contributions and approved this manuscript.

## Conflict of Interest Statement

The authors declare that the research was conducted in the absence of any commercial or financial relationships that could be construed as a potential conflict of interest.

## Supplementary Material

Table S1 Included genes in regularized regression with elastic net penalization.

## Abbreviations

PD, Parkinson’s disease; ICD, impulse control disorder; QUIP, Questionnaire for Impulsive-Compulsive Disorders in Parkinson’s Disease; SNP, single-nucleotide polymorphism; ROC, receiver-operating characteristics; AUC, area under the curve; DRT, dopaminergic replacement therapy; DA, dopamine agonist; PPMI, Parkinson’s progression markers initiative; UPDRS, Unified Parkinson’s Disease Rating Scale; LED, levodopa equivalent dose; MAF, minor allele frequency; EN, elastic net; LD, linkage disequilibrium.
