# Supplementary material for: Dopaminergic and Opioid Pathways Associated with Impulse Control Disorders in Parkinson’s Disease
Source: Front Neurol. 2018 Feb 28;9:109. doi: 10.3389/fneur.2018.00109 (PMC5835501; doi:10.3389/fneur.2018.00109)
Supplement: Table S1 — Included genes in regularized regression with elastic net penalization. [file Table_1.PDF]

Pathway	Gene	SNP	Pathway (...)	Gene (...)	SNP (...)
Dopmainergic			***Serotonergic***		
	DRD1	5326		DDC	11575375
	DRD2	35352421		DDC	11575457
	DRD2	6275		DDC	2876829
	DRD2	6277		DDC	4490786
	DRD3	6280		DDC	6950777
	DRD4	10902180		HTR2A	2296973
	DRD4	1870723		HTR2A	6312
	DRD4	7482904		HTR2A	6313
	DRD5	1967550		HTR2A	6314
	DRD5	1967551		SLC6A3	1042098
	DRD5	6283		SLC6A3	2245660
	ANKK1	1800497		SLC6A3	40358
	COMT	4646315		SLC6A3	429699
	COMT	4646318		SLC6A3	460000
	COMT	4680		SLC6A3	6347
	COMT	4818		SLC6A3	6876890
				SLC6A3	8179029
				SLC6A4	1042173
General				SLC6A4	6354
	BDNF	11030101		TPH2	1007023
	BDNF	11030102		TPH2	11178998
	BDNF	6265		TPH2	4290270
	BDNF	66866077		TPH2	7305115
	ADRA2C	7434444		TPH2	76143560
Glutamatergic			***Opioid***		
	GRIN2B	10772698		OPRK1	11992022
	GRIN2B	11055581		OPRK1	7815824
	GRIN2B	16909218		OPRK1	702764
	GRIN2B	16909222		OPRM1	1799971
	GRIN2B	1805482		OPRM1	2272381
	GRIN2B	1805522		OPRM1	34427887
	GRIN2B	1806201		OPRM1	650245
	GRIN2B	3026159		OPRM1	675026
	GRIN2B	3026160		OPRM1	677830
	GRIN2B	7301328		OPRM1	7341264
				OPRM1	7759388
				OPRM1	9282821
				OPRM1	9478525
				OPRM1	9479798
